# Impact of hand hygiene product accessibility on 5 moment compliance within an acute care facility ward

**DOI:** 10.1186/2047-2994-4-S1-P298

**Published:** 2015-06-16

**Authors:** DP Limbert, M Nabuurs-Franssen, P Besselink-Quint, F van den Wildenberg, J Schaafstra, J Hines, A Voss

**Affiliations:** 1 Research & Development, Deb Group Ltd, Denby, UK; 2 Department of Medical Micriobiology and Infectious Diseases, Canisius-Wilhelmina Hospital, Netherlands; 3 Department of Surgery, Canisius-Wilhelmina Hospital, Netherlands; 4 Department of Urology, Canisius-Wilhelmina Hospital, Netherlands

## Introduction

24/7 electronic monitoring of hand hygiene (HH) product use enables the accurate assessment of HH compliance and behavioural patterns in a facility ward pre and post intervention. One such intervention being an increase in HH product accessibility.

## Objectives

Assess the impact on HH behaviour of adding ABHR at point of patient care.

## Methods

A group HH surveillance system was installed in a surgery ward such that all existing wall mounted Soap & ABHR dispensers were monitored. Dispensers were located at the entrance to patient rooms and over the sinks within. Data was not made visible to the ward staff to ensure that behaviours were unaffected.

Following a baseline period of 70 days, portable ‘point-of-care’ ABHR dispensers were added at the end of the patient beds and the impact on HH compliance and dispenser usage patterns was assessed over a further 70 day intervention period.

## Results

During the baseline period the average daily calculated compliance was 23% (compared to an average of 65% in the presence of an observer).

During the intervention period the average daily calculated compliance was 26%

Analysis of the 13% increase in HH compliance revealed a reduction of 2% in HH events taking place in the hallway compared with an in-patient room increase of 34%.

## Conclusion

The work demonstrates that positive change in group behaviour can be affected by improved HH product access, with both an increase in overall HH compliance and a shift in the number of HH events taking place within the patient room, consistent with WHO 5 Moments teaching.

## Disclosure of interest

None declared.

